# Histopathologic changes associated with Meibomian Gland dropout in a dog

**DOI:** 10.1111/vop.12754

**Published:** 2020-03-18

**Authors:** Yasunari Kitamura, Reiko Arita, Yukihiro Miwa, Hiroko Iwashita, Akihiko Saito

**Affiliations:** ^1^ Yakumo Animal Hospital Hokkaido Japan; ^2^ Itoh Clinic Saitama Japan; ^3^ Department of Ophthalmology Keio University Tokyo Japan; ^4^ Triangle Animal Eye Clinic Tokyo Japan

**Keywords:** dog, dropout, histopathology, meibomian gland

## Abstract

**Purpose:**

To perform histopathologic analysis of tissue manifesting meibomian gland dropout on noncontact infrared meibography in a dog.

**Methods:**

A 14‐year‐old intact male Cairn terrier was evaluated at Triangle Animal Eye Clinic for dense corneal opacity of the right eye. A complete ocular examination was performed, including slit‐lamp biomicroscopy, tonometry, and noncontact meibography. Pigmentary glaucoma with elevation of intraocular pressure was diagnosed, and meibography revealed morphological changes suggestive of gland dropout in the middle of the upper right eyelid.

**Results:**

The globe was enucleated by the transpalpebral method, and palpebral tissue was subjected to histopathologic analysis. The analysis revealed an almost complete loss of meibomian gland structure accompanied by slight enlargement and proliferation of fibroblasts as well as by infiltration of plasma cells and lymphocytes.

**Conclusions:**

Meibomian gland dropout as detected by meibography can be associated with chronic inflammation.

## 1 INTRODUCTION

Meibomian glands, also known as tarsal glands, are sebaceous glands located within the margins of the upper and lower eyelids. Their primary function is to produce oily components of the lipid layer of the tear film, with these components being secreted along the margins of the eyelids. The lipid layer of the tear film facilitates smooth blinking and attenuates the evaporation of lacrimal fluid. Meibomian gland dysfunction (MGD)^1^, ^2^ is one of the main causes of lipid layer deficiency and evaporative dry eye in humans,^3^ with its diagnosis being based on examination of gland morphology and function. Meibography is applied to examine meibomian gland morphology, with the introduction of noncontact infrared meibography (NIM) systems having facilitated the widespread clinical adoption of this procedure.^4^ The observation of MGs by NIM is based on illumination of the tarsal plate with infrared light. NIM reveals meibomian glands of normal eyes as white areas. It is thought that the meibomian glands appear white as a result of the presence of the meibomian gland lipid. Findings of abnormal meibomian gland morphology by NIM include dropout, shortening, disruption, curvature, and expansion.^5^, ^6^, ^7^ Dropout is a finding that implies the partial or total loss of glandular tissue, and it is one of the morphological changes of the meibomian gland observed in diseases of the ocular surface such as dry eye and systemic diseases.^8^, ^9^, ^10^, ^11^ However, given that there are few opportunities to collect eyelid tissue from patients with MGD, histological testing of dropout sites has been rarely performed. We previously performed a histological investigation of morphological changes of meibomian glands detected by NIM in dogs.^12^ Here, we present a case of localized meibomian gland dropout as revealed by NIM in the center of the right upper eyelid in a Cairn terrier with pigmentary glaucoma. Given that the animal had lost the ability to see in the right eye and the globe was enlarged, enucleation including resection of the upper and lower eyelids was performed. The findings of the histopathologic analysis of meibomian glands in the resected tissue could thus be compared with those of the meibography.

## 2 CASE REPORT

**Figure 1:**
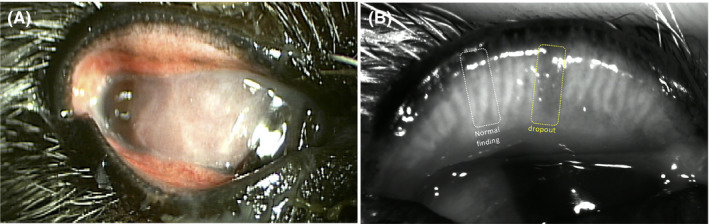
Appearance of the right eye. A, An ophthalmologic finding included a mucoid discharge, mild hyperemia of the upper and lower conjunctiva. Corneal vascularization and pigmentation with a corneal ulcer are apparent. B, Noncontact infrared meibography showed obvious gland dropout (yellow dashed box) and normal glands (white dashed box) in the middle of the upper eyelid

A 14‐year‐old intact male Cairn terrier was brought to Triangle Animal Eye Clinic for ophthalmologic testing of the right eye. General ophthalmologic findings included mucoid ophthalmic discharge, mild hyperemia of the upper and lower conjunctiva, eyelid swelling, extensive corneal pigmentation, and dense vascularization with corneal ulcer (Figure 1A). Corneal cytology revealed numerous neutrophils and some corneal epithelial cells. The intraocular pressures were estimated with a rebound tonometer (Tonovet; Tiolat, Helsinki, Finland) and were 51 and 12 mm Hg in the right and left eye, respectively. NIM with an Ocular Surface Analyzer (SBM, Torino, Italy) revealed local gland dropout in the center of the upper eyelid (Figure 1B), with no gland orifice being apparent in this region. The dog was diagnosed with chronic pigmentary glaucoma on the basis of ophthalmologic testing. Given that recovery of vision was judged to be unlikely and in order to relieve ocular discomfort, we selected enucleation as the appropriate treatment modality. The surgery included resection of the upper and lower eyelids, which were subjected to histopathological assessment after immersion fixation in 10% formalin.

**Figure 2:**
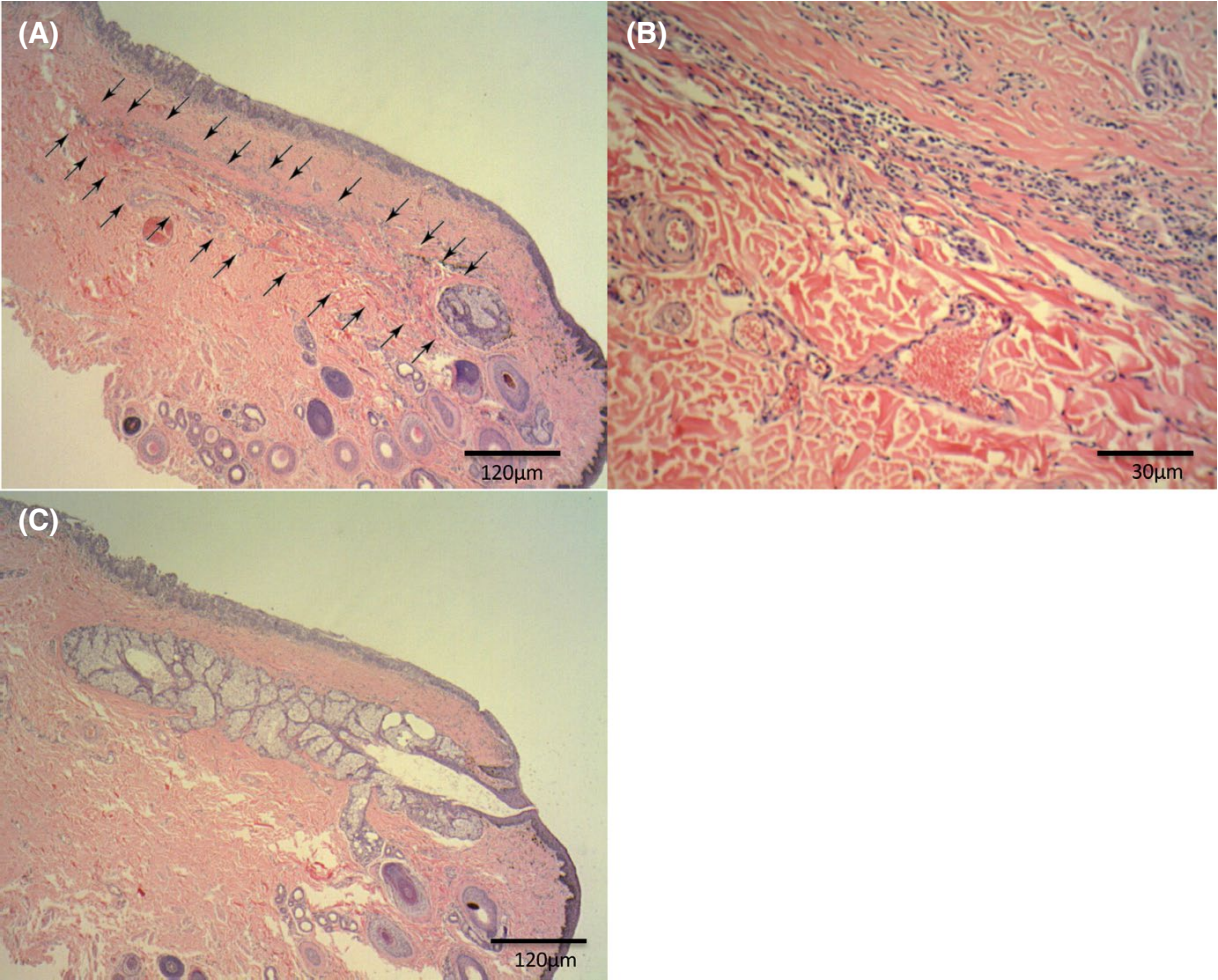
Hematoxylin‐eosin staining of resected eyelid tissue revealed the absence of normal gland lobular and ductal structure at the dropout site (arrows). Original magnification: ×100 (A) or × 400 (B). The histology of tissue corresponding to normal glands on meibography revealed a normal lobular structure of meibomian glands (C)

Histopathologic analysis of the tissue area corresponding to the site of gland dropout detected by NIM revealed meibomian gland atrophy with minimal lobular tissue remaining near the duct (Figure 2A). Chronic inflammation characterized by slight enlargement and proliferation of fibroblasts as well as by infiltration of plasma cells and lymphocytes was also apparent at this site (Figure 2B). The histology of tissue corresponding to normal regions on meibography revealed meibomian gland atrophy (Figure 2C).

## 3 DISCUSSION

The most severe finding of meibography is gland dropout, which is characterized by an apparent complete loss of gland structure. Sites of gland dropout appear dark on meibographic images, likely as a result of the loss of the oily material produced by meibomian glands. In the present case, histopathology revealed the loss of lobular and ductal tissue at the site of gland dropout. This site included chronic inflammation, characterized by the presence of slightly enlarged and proliferating fibroblasts and by infiltration of plasma cells and lymphocytes. Although this inflammatory response may have been the result of a foreign body reaction to oil remaining in the meibomian gland duct or of the effects of ocular surface inflammation on the eyelid margin, its cause could not be discerned from our histological findings.

We previously performed histopathologic analysis at the site of meibomian gland dropout in a 15‐year‐old miniature dachshund without evident ophthalmologic disease. The results revealed destruction of the lobular and ductal structure of the gland and multiple vacuolar lesions in connective tissue not accompanied by inflammatory cell infiltration.^12^ In the present case, the histopathologic findings were markedly different, being characterized by chronic inflammation. It is difficult to determine the cause of gland atrophy from the histopathologic changes in the present case as well as to provide insight into the relation between the present histopathologic findings and those of our previously reported case. However, one finding that was common to the dropout site in both cases was the destruction of the lobular and ductal structure of the gland. Eom et al reported on the correlation between tear film lipid layer and meibomian gland dropout of patients with meibomian gland dysfunction (MGD).^13^ The lipid layer thickness was negatively correlated with meibomian gland loss. It is therefore likely that the detection of extensive gland dropout affecting more than 30 percent of the eyelid by NIM corresponds to a marked decrease in the production and secretion of oil by meibomian glands and a subsequent attenuation of the tear film lipid layer thickness. Future histopathologic testing of tissue from sites of various other morphological changes detected by NIM, such as shortening and curvature, will be necessary to elucidate the histological changes that precede gland dropout. Such analysis may also shed light on the etiology and pathogenesis of MGD and other meibomian gland‐related diseases and thereby provide a basis for the development of new treatments.

## CONFLICTS OF INTEREST

None declared.
